# Confirming the geography of fatty infiltration in the deep cervical extensor muscles in whiplash recovery

**DOI:** 10.1038/s41598-020-68452-x

**Published:** 2020-07-10

**Authors:** Andrew C. Smith, Stephanie R. Albin, Rebecca Abbott, Rebecca J. Crawford, Mark A. Hoggarth, Marie Wasielewski, James M. Elliott

**Affiliations:** 1 School of Physical Therapy, Regis University, Denver, CO USA; 2 Physical Therapy and Human Movement Sciences, Feinberg School of Medicine, Northwestern University, Chicago, IL USA; 3 Department of Mechanical Engineering, Northwestern University, Evanston, IL USA; 4 Body Urbanist, Rotterdam, South Holland, The Netherlands; 5 Department of Biomedical Engineering, Northwestern University, Evanston, IL USA; 6 Faculty of Medicine and Health, The Northern Sydney Local Health District, The Kolling Institute, The University of Sydney, St. Leonards, NSW 2065 Australia

**Keywords:** Skeletal muscle, Anatomy, Musculoskeletal system, Skeleton, Prognostic markers

## Abstract

Previous preliminary work mapped the distribution of neck muscle fat infiltration (MFI) in the deep cervical extensor muscles (multifidus and semispinalis cervicis) in a small cohort of participants with chronic whiplash associated disorders (WAD), recovered, and healthy controls. While MFI was reported to be concentrated in the medial portion of the muscles in all participants, the magnitude was significantly greater in those with chronic WAD. This study aims to confirm these results in a prospective fashion with a larger cohort and compare the findings across a population of patients with varying levels of WAD-related disability one-year following the motor vehicle collision. Sixty-one participants enrolled in a longitudinal study: Recovered (n = 25), Mild (n = 26) and Severe WAD (n = 10) were studied using Fat/Water magnetic resonance imaging, 12-months post injury. Bilateral measures of MFI in four quartiles (Q1–Q4; medial to lateral) at cervical levels C4 through C7 were included. A linear mixed model was performed, controlling for covariates (age, sex, body mass index), examining interaction effects, and comparing MFI distribution between groups. The recovered group had significantly less MFI in Q1 compared to the two symptomatic groups. Group differences were not found in the more lateral quartiles. Results at 12 months are consistent with the preliminary study, indicating that MFI is spatially concentrated in the medial portions of the deep cervical extensors regardless of WAD recovery, but the magnitude of MFI in the medial portions of the muscles is significantly larger in those with severe chronic WAD.

## Introduction

Various investigations across three continents (United States^1–3^, Australia^2,4–6^, and Sweden^7,8^) have used similar manual segmentation methods with magnetic resonance imaging (MRI) to demonstrate widespread fatty infiltrates in the neck muscles of individuals with acute and chronic whiplash associated disorders (WAD). Comparably high levels of muscle fat infiltration (MFI) were not identified in individuals who (i) had recovered or reported lower levels of WAD-related disability^1,3,4,7,8^, (2) had chronic non-traumatic neck pain^9^, or (3) those without a history of neck disorders^10^. Knowledge of a precise mechanism for MFI expression and distribution (e.g. extra- or intra-fascicular MFI) would provide a target for clinical remediation of any adverse symptoms; but this is largely unknown.

There remains conjecture in the literature about what constitutes extra- or intra-fascicular MFI, which likely centres on our ability to reliably distinguish the epimysium/perimysium and associated muscle fascicles on MRI. Some have proposed standardised, anatomical definitions of regions of interest in quantifying spinal muscle composition^11,12^, which we concede can be difficult for the cervical spine (eg transversospinal muscles like multifidus).

With this in mind, total MFI has been observed and reported to occur throughout cervical spine levels with a greater magnitude of MFI occurring amongst the deepest muscular layer of the extensors (e.g. comprising the cervical multifidus and semispinalis cervicis) when compared with the more superficial musculature (e.g. including semispinalis capitis, splenius capitis and upper trapezius)^10,13^. In earlier preliminary work, Abbott et al.^1^ reported that, in a small cohort of participants with chronic WAD and healthy controls, the composition of the deep cervical muscles appeared dependent on location in the transverse plane where the most medial muscle tissues approximating the spinous processes have larger proportions of MFI.

It is noteworthy that while MFI was reported to be more concentrated in the medial portion of the muscles in all participants^1,7^, the magnitude was significantly greater in a small subgroup of participants with chronic WAD, suggesting a potential phenotypic expression of chronicity. Despite positive findings from both quantitative^1^ or qualitative^7^ measures, replication of these findings in a larger cohort with varying levels of chronic WAD is required before the pervasive finding of, and specific distribution for, MFI in this population can be confirmed. As such, MRI data from participants enrolled in a longitudinal study of WAD recovery were included from the 1-year post injury time point and used to (i) confirm previous results^1^ by quantifying the magnitude and spatial distribution of MFI in the deep cervical extensor muscles (multifidus and semispinalis cervicis) and (ii) compare these findings across a population of patients with varying levels of WAD-related disability one-year following the injury event.

## Methods

### Sub-study population

Sixty-one participants, out of a total 97, were enrolled in this sub-study of a prospective, longitudinal parent study investigating the neuromuscular mechanisms underlying poor recovery following whiplash injury from a motor vehicle collision (MVC) (ClinicalTrials.gov (Identifier: NCT02157038)). The study was approved by Northwestern University’s Institutional Review Board. All applicable institutional and governmental regulations concerning the ethical use of human volunteers were followed during the course of this research according to the Declaration of Helsinki, and written informed consent was obtained from every participant.

**Figure 1:**
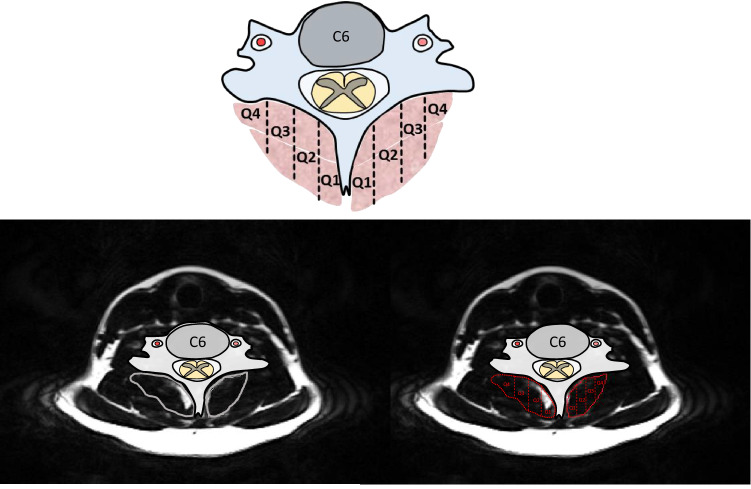
Deep cervical extensor muscle segmentation was delineated into the four quartiles, with Q1 being most medial (near the spinous process) and Q4 most lateral.

After an MVC, participants were asked to participate through the university-affiliated emergency medicine department. Inclusion criteria were: neck pain following an MVC, within the Quebec Task Force Classification category of WAD Grade II (primary neck pain complaint, reduced neck range of movement and point tenderness in the neck, with no radicular symptoms), and completed both the questionnaires and magnetic resonance imaging (< 1-week, 2-weeks, 3-months, and 12-months post MVC). Exclusion criteria were: < 18 or > 65 years of age at time of collision, prior motor vehicle collisions, treatment for neck pain disorders within the past 10 years, neurological or metabolic disorders (Multiple Sclerosis, Parkinson’s, Alzheimer’s, or diabetes), or at risk for poly-trauma (determined by emergency department protocols). A total of 61 participants were included having the available MRI data at 12-months post injury that was suitable for quartile segmentation in the transverse plane, with Q1 being most medial near the spinous process and Q4 most lateral, employing a method previously described^1^ and shown in Fig. 1. Neck Disability Index scores at one-year post injury were used to determine severity groups: (1) severe group (≥ 30%), (2) mild group (10–29%), and (3) recovered group (< 10%)^3,4^.

### Magnetic resonance imaging

**Figure 2:**
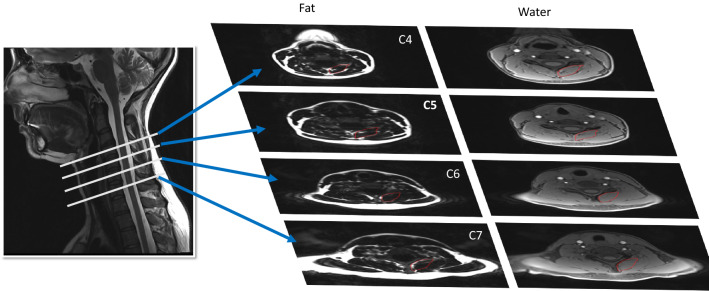
Fat/Water MRI of the deep cervical extensors from C4 through C7.

Each participant received MRI of their deep cervical extensors from C4 through C7 (Fig. 2), using a 3.0 T scanner with a 64-channel head/neck coil (Siemens, Munich Germany). Specifics of the fat/water MRI were as follows: dual-echo gradient-echo sequence (2-point Dixon), TR = 7.05 ms, TE1 = 2.46 ms, TE2 = 3.69 ms, flip angle = 12°, bandwidth = 510 Hz/pixel, FOV = 190 × 320 mm^2^, slab oversampling of 20% with 40 partitions to prevent aliasing in the anterior–posterior direction, in-plane resolution = 0.7 × 0.7 mm^2^, slice thickness = 3.0 mm^3^, number of averages = 6, acquisition time = 4 min 5s^14^. This fat/water sequence has been validated in animal models^15^.

The deep cervical extensors (multifidus and semispinalis cervicis) were manually segmented^12^ according to an established method previously described^1^. Briefly, this approach was shown to have high inter- and intra-rater reliability^1^. MFI was calculated in the same fashion as the previous cross-sectional study using an average of three slices per vertebral level: Muscle Fat Infiltration = I_F_/(I_F_ + I_W_) × 100, where I_F_ = fat signal intensity and I_W_ = water signal intensity^1^. Deep cervical extensor muscle segmentation was delineated into the four quartiles, with Q1 being most medial (near the spinous process) and Q4 most lateral, using a custom MATLAB script (The MathWorks, Inc, Natick, MA) (see Fig. 1).

### Statistical analysis

SPSS Version 23.0 was used to perform all statistical analyses (IBM Corporation, Armonk, NY). Baseline descriptive statistics were summarized and assessed for potentially important demographic differences. A repeated-measures, linear mixed-model approach was used to investigate MFI differences between recovery groups. The main and interactive effects of muscle quartile and group (severe, mild, and recovered) were modelled as fixed effects, and each cervical level was analyzed utilizing a separate mixed model. Inter-participant differences in the change in MFI across quartiles were modelled as random effects. Age, sex, and body mass index were entered as covariates into each model as per our primary objective of confirming previous work in which these covariates were used^1^. Pairwise comparisons were used to investigate between-group differences in intra-quartile MFI. For all analyses, the significance level was set to *p* < 0.05.

## Results

**Figure 3:**
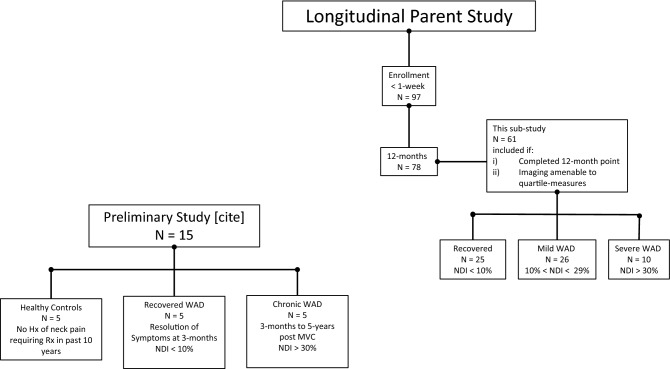
Flowchart of participants involved in parent study, sub-study, and previous preliminary work.

A total of 97 acutely injured participants were enrolled in our parent study and followed for four time points (< 1-week, 2-weeks, 3-months, and 12-months post MVC). All participants were followed up for the first three time points, but a total of 19 were lost to attrition between 3-months and 12-months. Thus, a total of 78 returned for the 12-month follow-up. Of this 78, we had available quartile imaging data for each time point (< 1 week, 2 weeks, 3 months and 12 months) in a total of 61 participants, which leaves a total of 17 participants without such data; one of which was due to poor scan quality that was not amenable to quartile measures. The remaining 16 were not included (severe = 5; mild = 6; recovered = 5) on grounds they did not have imaging that was amenable for quartile segmentation in the transverse plane (see Fig. 3).

A total of 10 participants were assigned to the severe group, 26 to the mild group, and 25 to the recovered group (N = 61 for all participants, see Table 1 including comparison to the Severe and Recovered participants from Abbott et al.^1^) based on NDI % scores at 12 months post-MVC. Table 2 details group descriptive data at 12-months post whiplash for those in, and those not in, this sub-study investigating quartile MFI.

**Table 1:** Participant Demographics and comparison to Abbott et al.^1^ participants.

Characteristic	Severe N = 10	Severe N = 5 Abbott et al.^1^	Mild N = 26	Recovered N = 25	Recovered N = 5 Abbott et al.^1^
BMI, kg/m^2^	25.7 ± 2.8	29.6 ± 24.6	23.8 ± 3.7	23.6 ± 3.1	26.4 ± 1.1
Age, y	37.0 ± 12.5	30.6 ± 9.0	37.9 ± 12.5	31.5 ± 10.7	32.8 ± 7.5
Sex (female), n (%)	8 (80)	3 (60)	23 (88)	14 (56)	3 (60)

Values are mean ± SD unless otherwise indicated.

*BMI* body mass index.

**Table 2:** Additional participant demographics (from parent study, not included).

Characteristic	Not included	Included	Not included	Included	Not included	Included
	Severe N = 5	Severe N = 10	Mild N = 6	Mild N = 26	Recovered N = 5	Recovered N = 25
BMI, kg/m^2^	28.0 ± 7.3	25.7 ± 2.8	25.7 ± 6.1	23.8 ± 3.7	26.6 ± 2.8	23.6 ± 3.1
Age, y	36.1 ± 9.1	37.0 ± 12.5	27.9 ± 9.3	37.9 ± 12.5	32.8 ± 7.3	31.5 ± 10.7
Sex (female), n (%)	4 (80)	8 (80)	5 (83)	23 (88)	3 (60)	14 (56)
NDI	29.2 ± 19.2	31.8 ± 11.6	17.6 ± 16.1	19.5 ± 8.7	2.8 ± 4.4	6.1 ± 6.7

Values are mean ± SD unless otherwise indicated.

*BMI* body mass index.

**Figure 4:**
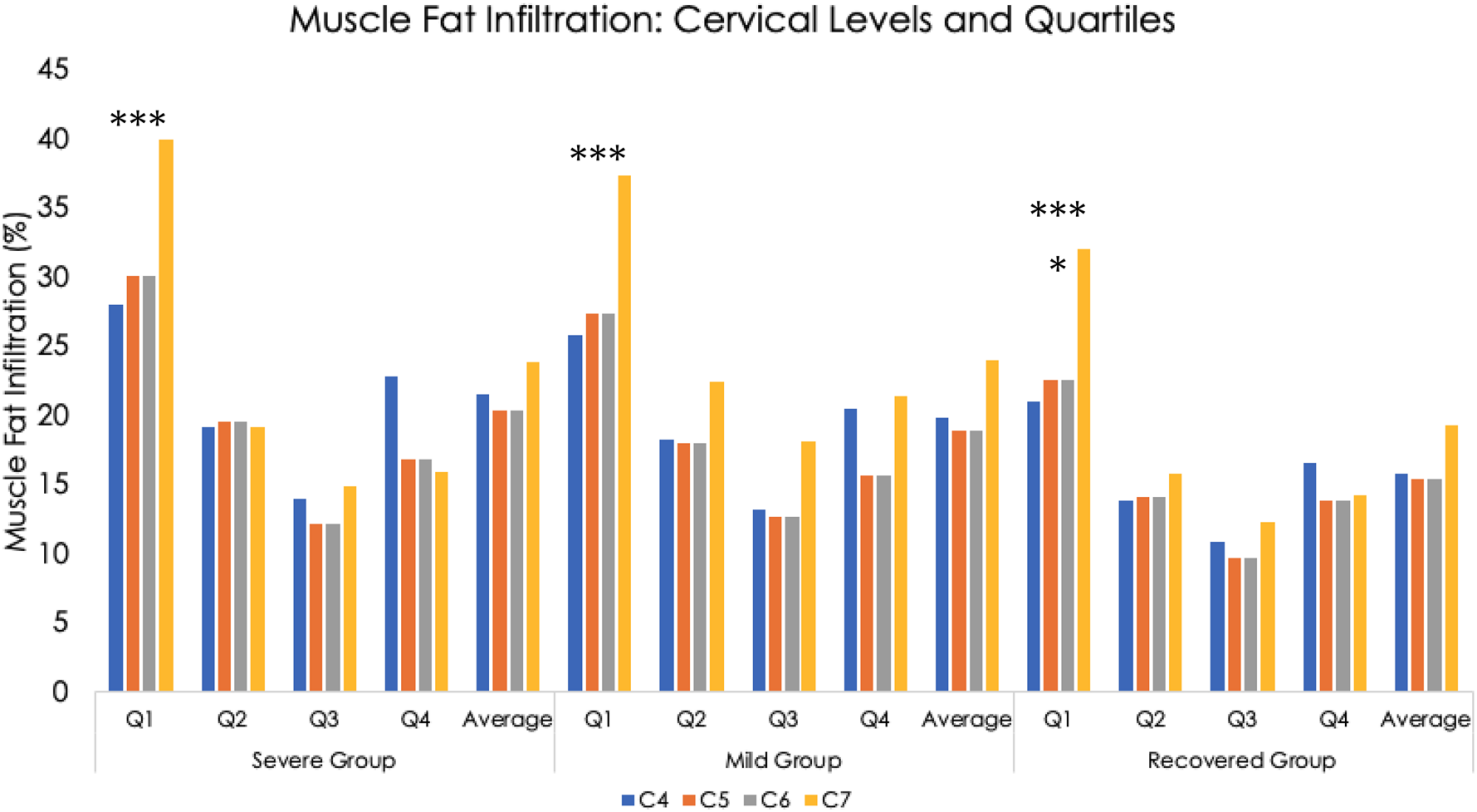
Muscle fat infiltration by group, Cervical level (C4–C7, Quartiles (Q1–Q4), and average of the all Quartiles*Level.

For all three groups, the most medial quartile, Q1, had the highest level of MFI (p < 0.001, see Fig. 4). For all four cervical levels (C4 through C7), the recovered group had significantly less MFI in Q1 compared to the symptomatic groups (*p* < 0.05). This was also found for the second-most medial quartile, Q2, at levels C4 and C5 (*p* < 0.05). Group differences were not found in the more lateral quartiles, Q3 and Q4 at any level (C4 through C7). There was a trend towards a significant group * quartile interaction effect at the cervical level, C5 (*p* = 0.05).

## Discussion

Consistent with preliminary work^1,7^, MFI appears to be equally distributed between the more lateral quartiles at all levels analysed (C4-C7) and across all groups, but, once again, Q1 contains the highest MFI levels with significantly larger magnitudes in the two symptomatic groups compared to the recovered group. This provides supportive data mapping the spatial distribution and magnitude of MFI in the deep extensor muscles in a larger sample of individuals with varying levels of WAD-related disability at one-year post injury when compared to preliminary quantitative^1^ and qualitative^7^ reports.

As a reflection of the natural history of decline in muscle quality with age, it is plausible that the medial aspect of deep paraspinal muscles is susceptible to increased MFI, thereby raising mechanistic questions of whether (i) having higher pre-trauma MFI in Q1 or (ii) the acute trauma-related expressions of Q1 MFI contribute to the clinical course and further degeneration in total MFI over time. Questions also remain regarding the influence that age, sex, BMI, stress and/or an inflammatory response, and injury severity, have on muscle composition and if certain cervical levels are more mechanically disadvantaged than others. For example, the C5/C6 disc has been found to be at highest risk of injury during both frontal and rear impact MVC’s^16^. In addition to the C5/C6 disc, others have demonstrated the intervertebral foramen at C5/C6 narrowed by as much as 1.8 mm during simulated rear impacts using cadaveric tissue^17,18^. It is proposed that such narrowing could compress the nerve roots and ganglia in the lower cervical spine, particularly in individuals with congenitally narrow foramen or those with pre-collision osteophytes. Recent evidence also provides foundation that the number of degenerative pathologies seen on initial post MVC computed tomography^19^, and pre-collision medical diagnoses^20^ may be associated with the subsequent clinical course of whiplash, and this could align with our findings of significantly larger magnitudes of Q1 MFI, notably at C5, in the symptomatic participants at 12-months. Comparing quartile MFI to the number of degenerative pathologies at all cervical levels across injured male and female participants of varying age is warranted and underway.

It is important to also recognize these findings support previous preliminary cross-sectional work involving patients with varying levels of chronic WAD-related disability and healthy controls across two cultures (United States^1^ and Sweden^7^); where different insurance schemes and medical/rehabilitative options may influence recovery. While larger proportions of MFI in the multifidus muscles have been attributed to severe WAD, further prospective investigations, with larger sample sizes, are required to identify an element of pre-existing organic origins^19–21^, specific injury mechanisms underlying MFI^22,23^, biomechanical consequences^24–28^ and their relationships to the further development of MFI with a long-term goal of informing clinical trials; exercise in particular^29^.

The deep cervical extensors (e.g. multifidus and semispinalis cervicis muscles) are architecturally complex, with Q1 fascicles attaching to the spinous process of a superior vertebra (e.g. C5) and then traveling laterally (Q2-Q4) to attach to the transverse process of an inferior vertebra (e.g. C6). While the specific biomechanical consequences of MFI distribution remain speculative, several hypotheses arise. If increases in local MFI have the potential to influence motor function^30^, the magnitude and distribution of MFI may be clinically important in traumatic neck pain conditions^31,32^ and towards informing biomechanical models of the human head/neck^33^. Despite an emerging body of evidence examining muscle compositional change, the magnitude and spatial distribution of MFI has received little attention in informing current modelling efforts. Further work in this domain appears warranted as has been documented for the lumbar spine^34,35^.

The multifidus and semispinalis cervicis share a common nerve supply with, and attach directly to, the zygapophyseal joint capsules. The latter have been widely implicated in the generation and maintenance of neck pain following a traumatic MVC^36–39^, with nearly half of those injured treated for, and receiving benefit from their, facetogenic symptoms^40–43^. Specifically, radiofrequency neurotomy (RFN) interventions have shown to attenuate the pervasive psychophysical signs/symptoms (e.g. thermal/pressure pain thresholds) common to chronic WAD^44^. However, in considering the procedure is frequently repeated (once the pain returns) in a large proportion of patients, the long-term biomechanical consequences of RFN and its effects on muscle structure, function, and long-term outcomes, are largely unknown, but could be the result of chronic inhibition of muscle tissue. Further exploration with current quantitative methods are warranted to better understand the long-term biomechanical and functional outcomes with repeated RFN procedures^45^.

Our study is not without limitations. Neck muscle composition may be influenced by an individual’s general activity and/or neck-specific exercise levels, particularly in people with persistent WAD where symptoms may limit participation^46,47^. Accordingly, future work should aim to capture general and task-specific activity data across a larger population of participants with varying levels of WAD recovery.

## Conclusions

These results provide confirmation for unique patterns of MFI distribution within the deep extensor muscles of participants with varying levels of WAD-related disability one year following the injury event. This confirms previous findings highlighting that the distribution of deep neck muscle MFI is not uniquely featured in those with poor recovery one-year following a whiplash injury from an MVC, but rather the total magnitude of MFI is greater, especially regarding the medial portions at Q1, in those with poor recovery.

Advanced MRI sequencing methods are showing promise in the lumbar spine in identifying fibre type^48^, which has previously only been possible with biopsied tissues. Muscle fibre ‘transformation’ or ‘replacement’ models remain controversial and certainly warrant further study. Future mechanistic work towards identifying an element of pre-existing organic origins to the natural history of MFI is required before definitive conclusions can be drawn. Such work will help in understanding if certain individuals have a propensity for higher MFI and if the magnitude and distribution of MFI results in biomechanical deficits and persistent WAD-related disability. The revelation of different recovery phenotypes and mechanistic/physiologic processes would likely inform clinical trials.
